# Renal Cortical Lactate Dehydrogenase: A Useful, Accurate, Quantitative Marker of *In Vivo* Tubular Injury and Acute Renal Failure

**DOI:** 10.1371/journal.pone.0066776

**Published:** 2013-06-18

**Authors:** Richard A. Zager, Ali C. M. Johnson, Kirsten Becker

**Affiliations:** 1 The Department of Medicine, University of Washington, Seattle, Washington, United States of America; 2 Clinical Division, Fred Hutchinson Cancer Research Center, Seattle, Washington, United States of America; University of Sao Paulo Medical School, Brazil

## Abstract

Studies of experimental acute kidney injury (AKI) are critically dependent on having precise methods for assessing the extent of tubular cell death. However, the most widely used techniques either provide indirect assessments (e.g., BUN, creatinine), suffer from the need for semi-quantitative grading (renal histology), or reflect the status of residual viable, not the number of lost, renal tubular cells (e.g., NGAL content). Lactate dehydrogenase (LDH) release is a highly reliable test for assessing degrees of in vitro cell death. However, its utility as an in vivo AKI marker has not been defined. Towards this end, CD-1 mice were subjected to graded renal ischemia (0, 15, 22, 30, 40, or 60 min) or to nephrotoxic (glycerol; maleate) AKI. Sham operated mice, or mice with AKI in the absence of acute tubular necrosis (ureteral obstruction; endotoxemia), served as negative controls. Renal cortical LDH or NGAL levels were assayed 2 or 24 hrs later. Ischemic, glycerol, and maleate-induced AKI were each associated with striking, steep, inverse correlations (r, −0.89) between renal injury severity and renal LDH content. With severe AKI, >65% LDH declines were observed. Corresponding prompt plasma and urinary LDH increases were observed. These observations, coupled with the maintenance of normal cortical LDH mRNA levels, indicated the renal LDH efflux, not decreased LDH synthesis, caused the falling cortical LDH levels. Renal LDH content was well maintained with sham surgery, ureteral obstruction or endotoxemic AKI. In contrast to LDH, renal cortical NGAL levels did not correlate with AKI severity. In sum, the above results indicate that renal cortical LDH assay is a highly accurate quantitative technique for gauging the extent of experimental acute ischemic and toxic renal injury. That it avoids the limitations of more traditional AKI markers implies great potential utility in experimental studies that require precise quantitation of tubule cell death.

## Introduction

There is a growing awareness of the importance of acute kidney injury (AKI) as a major contributing factor to death in critically ill patents [1]–[4], and to the possible onset of progressive renal disease [5]–[10]. These facts have fostered the growth of a burgeoning number of experimental studies to interrogate cellular injury pathways that potentially can be targeted, with the goal of preventing AKI and its consequences.

A critical need in such studies is the ability to precisely define the severity of kidney injury in order to judge the efficacy of potential therapeutic agents. The vast majority of studies utilize BUN and plasma creatinine as surrogate markers of glomerular filtration, and hence, overall injury severity. However, BUN and creatinine assessments can suffer from serious shortfalls. Notable are the following: first, GFR is not a direct assessment of tubular injury, and changing intrarenal hemodynamics can obfuscate results. Second, non renal events can independently alter BUN and creatinine levels (e.g., urea overproduction in hypercatabolic states; excess creatinine generation with rhabdomyolysis). Third, concomitant volume depletion, as frequently occurs in the setting of experimental AKI (e.g., due to surgical fluid losses, or poor fluid intake), can induce a pre-renal state, such that BUN and creatinine elevations over-estimate the severity of renal damage; Fourth, fixed rates of BUN and creatinine generation can limit the degree to which increases can occur. Thus, with very severe renal injury, BUN and creatinine values will no longer parallel the extent of renal damage. And fifth, with unilateral AKI studies, e.g., unilateral renal ischemia, a normal contralateral kidney prevents BUN and creatinine increases.

A frequently used alternative, or supplemental, approach to BUN and creatinine assessments is the evaluation of renal histology. However, there are also substantial limitations with this approach. First, there is a significant time delay between the onset of tubular injury and the microscopic appearance of overt morphologic damage. Thus, the primary utility of histologic assessments is during the late injury phase (e.g. 24 hrs following ischemic or toxic renal damage). Second, most histologic assessments of AKI are performed on immersion fixed kidney sections. However, immersion fixation induces a host of artifacts (e.g., tubule vacuolization, brush border damage). Thus, if renal histology is to be relied upon for making renal injury assessments, technically challenging in vivo perfusion fixation should ideally be performed. Third, renal histologic changes, e.g., as induced by ischemic renal injury, are notoriously patchy in their presentation, with areas of severe necrosis adjacent to areas which demonstrate near normal histology. Finally, assessments of renal histology typically use highly subjective, operator dependent, “semi-quantitative” scales to grade injury severity. As such, there is essentially no uniformity between different laboratories.

A third approach to quantifying AKI severity is to measure “stress proteins” that are up-regulated in response to AKI. Such markers include heat shock proteins, NGAL, KIM-1, or injury- induced cytokine/chemokine levels. However, two major drawbacks compromise this approach. First, the above “biomarker” proteins are low molecular weight, and hence, they can undergo glomerular filtration and tubular reabsorption. Thus, extra renal production can cloud interpretation of renal tissue levels (as will be illustrated in this report). Second, as stress reactants, these proteins basically reflect production by residual viable cells, and not the cells that have been lost to necrotic or apoptotic cell death. Hence, these stress protein markers, by their very nature, cannot provide a direct and accurate index of tubular cell loss.

Given the above limitations, and given the importance of being able to accurately assess cell death in response to AKI, we have tested a new hypothesis: that lactate dehydrogenase (LDH) loss from the kidney might provide an accurate index of the extent of tubular cell death. Noteworthy in this regard is that LDH release is widely used as a definitive marker of cell death in cell culture [e.g. 11,12] and isolated proximal tubule segment [13] experiments. In vitro utility is made possible because intracellular LDH is rapidly released from severely damaged cells into the cell culture media. However, whether LDH release can readily occur from an *intact* kidney (e.g., making its way out of the interstitium or tubular lumina), and whether potential renal LDH losses can provide an *accurate quantitative index of renal injury severity*, have not previously been defined. Indeed, if the latter were the case, then renal tissue LDH content could serve as a highly valuable adjunct for assessing AKI injury, and thereby complement more traditional (BUN, creatinine, histology) AKI assessments. This study was undertaken to address this possibility. We then go on to demonstrate the utility of this approach by studying the phenomenon of aging effects on renal susceptibility to ischemic and toxic proximal tubule damage.

## Methods

All experiments were performed using 30–45 gm male CD-1 mice (Charles River Laboratories, Wilmington, MA) maintained under standard vivarium conditions. This study was carried out in strict accordance with the recommendations in the Guide for the Care and Use of Laboratory Animals of the National Institutes of Health. The protocol was approved by the Committee on the Ethics of Animal Experiments of the Fred Hutchinson Cancer Research Center (Permit Number: #A3326-1103). All surgery was performed under sodium pentobarbital anesthesia (40–50 mg/Kg IP), and all efforts were made to minimize suffering.

### Variable Unilateral Renal Ischemia Times/18 hr Recovery

To assess the relationship between variable renal ischemia times and degrees of potential renal LDH losses, 18 mice were subjected to a midline laparotomy, the left renal pedicle was identified, and then occluded with an atraumatic vascular clamp. After either 15 min, 22 min, 30 min, 40 min, or 60 min of ischemia (n, 3, 4, 4, 4, and 3 mice respectively), the clamps were removed, the abdominal incisions were closed in two layers, and the mice were allowed to recover from anesthesia. Body temperature was maintained at 37°C throughout the ischemia/early reperfusion period with external heating sources. Free food and water access was provided. At 18 hrs post ischemia, the mice were re-anesthetized, a blood sample was obtained from the inferior vena cava, and then the post ischemic and contralateral kidneys were resected. Both renal cortical and whole kidney tissue sections were cut and extracted for protein and total RNA. LDH content was determined by enzymatic assay (Promega, Madison, WI G1782). As an index of LDH gene transcription, LDH–A and LDH–B mRNAs (the two mRNAs that encode the two subunits of LDH-4, the dominant isoform within kidney) were assessed by RT-PCR, using the primers presented in Table 1. Results were expressed as a ratio to simultaneously obtained GAPDH product. In addition, 5 kidneys obtained from normal mice (not previously subjected to surgery) were harvested to provide control renal cortical and plasma samples.

**Table 1:** Mouse primers used to quantify renal cortical LDH subunit mRNAs.

mRNA	Primer Sequences	Product Size
LDH-A	5′-TGG AAG ACA AAC TCA AGG GCG AGA-3′ 5′-TGG ATT GGA GAC GAT CAG CAG CTT-3′	257 bp
LDH-B	5′-AGC ATT CTG GGA AAG TCT CTG GCT-3′ 5′-TGT CCA CCA CCA TCT TAT GCA CCT-3′	598 bp
GAPDH	5′-CTG CCA TTT GCA GTG GCA AAG TGG-3′ 5′-TTG TCA TGG ATG ACC TTG GCC AGG-3′	437 bp

Mouse primer pairs used for detection of the mRNAs for the LDH-A and LDH-B subunits of LDH-4 (the dominant renal LDH isoform). Results were expressed as a ratio to simultaneously obtained GAPDH product.

### Variable Bilateral Ischemia Times/18 hr Reflow: Renal Tissue, Plasma, and Urinary Assessments

To assess the degree to which variable bilateral ischemia times impacts tissue LDH content and BUN concentrations, and to determine whether LDH gains urinary and plasma access, 6 mice were subjected to bilateral ischemia of either 15 min, 30 min or 45 min duration (n, 2 each), using the surgical protocol presented above. Eighteen hours later, the abdominal incision was opened, a urine sample was obtained by bladder compression, a blood sample was collected from the inferior vena cava and then both kidneys were resected. Plasma samples were assayed for BUN and LDH concentrations; the urine samples were assayed for LDH (expressed as a ratio to the urinary creatinine concentrations); and bilateral renal cortical tissue protein extracts were assayed for LDH content. The potential variation in left vs. right kidney LDH levels following a given amount of ischemia was assessed.

### Variable Unilateral Ischemia Times/2 hrs Reflow

To determine whether renal cortical LDH reductions might be an early marker of injury (i.e., before the onset of histologic evidence of tissue necrosis, or before BUN elevations would be expected to occur), 12 mice were subjected to variable lengths of left renal ischemia (0 min, 20 min, 40 min, 60 min; n, 4, 2, 4, and 2 respectively) and they were maintained under anesthesia for 2 hrs of reflow. At the two hr time point, plasma samples were obtained for LDH assay, and then the kidneys were extracted. Renal cortical samples were used for LDH analysis.

### Assessment of whether Degrees of Non Ischemic forms of Tubular Necrosis can be Quantified by Decreases in Renal Cortical LDH Concentrations

Four non ischemic AKI models were chosen: 1) maleate nephrotoxicity (n, 5 mice; 600 mg/Kg by IP injection); [14]; 2) the glycerol model of rhabdomyolysis induced AKI (n, 5 mice; 8 mg/Kg of 50% glycerol injected in equally divided doses into the hind limbs) [15]; 3) left unilateral ureteral obstruction (n, 5 mice, ureter ligated at its midpoint with a silk suture applied through a midline abdominal incision) [15]; and 4) or endotoxemia (n, 4 mice; 2 mg/Kg E coli LPS) [25]. Eighteen hrs later, the mice were anesthetized and renal cortical samples were obtained for LDH assay. Samples from 5 normal mice served as controls. The severity of renal injury was assessed by measuring terminal BUN concentrations. Plasma/kidney samples from 5 normal mice served as controls.

### Renal Cortical NGAL Assessments; Comparison to Renal Cortical LDH Results

NGAL is a widely used biomarker of AKI and its severity. Hence, NGAL assay was performed on the renal samples obtained in the previously described unilateral ischemia/18 reflow experiments, using a commercially available NGAL ELISA kit (R&D Systems, Minneapolis, MN; DY 1857). The NGAL vs. LDH results were then compared.

### Testing the Potential Research Application of Renal Cortical LDH Content

There has been a growing literature which indicates that advancing age imparts increasing susceptibility to ischemic and nephrotoxic AKI [16]–[18] **.** However, whether these differences reflect differences in renal/intraglomerular hemodynamics (as previously suggested) [17], or whether proximal tubules become more susceptible to injury with advancing age, remains unknown. Hence, we used renal cortical LDH content following ischemic AKI and a nephrotoxic AKI model (glycerol induced rhabdomyolysis) to test whether LDH tissue assay can provide new insights in this regard.

#### Ischemic renal injury

Male CD-1 mice of either 2, 6, or 12 months of age (n, 4 at each age) were subjected to 30 min of left unilateral ischemic injury, as described above. Eighteen hrs later, both kidneys were resected and the renal cortices were assayed for LDH. For comparison, tissue NGAL levels were assessed. [Note: the unilateral rather than the bilateral ischemia model was chosen because the contralateral kidney could provide a baseline index of tissue LDH content, which could potentially be altered by advancing age].

To provide an index of histologic injury, transverse renal sections were cut, fixed in formalin, and 2 micron paraffin sections were stained with hematoxylin and eosin. The slides were blind coded, and evaluated for the extent of tubular necrosis using a semi-quantitative scale of 1–6 (least to most extensive injury observed).

#### Glycerol induced renal injury

To further assess whether age impacts the severity of AKI, and whether renal cortical LDH has utility as a marker of this process, 2 month and 12 month old mice (n, 4 each) were injected with 8 ml/Kg of glycerol, as described above. Eighteen hrs later, the severity of renal injury was assessed by BUN and plasma creatinine concentrations and by the level of renal cortical LDH. Because heme oxygenase levels are a critical determinant of heme protein toxicity [19]–[21], both HO-1 and HO-2 mRNAs and HO-1 protein levels were assessed. Because plasma HO-1 protein levels can serve as an index of AKI severity [22], it’s concentrations in terminal plasma samples were also measured (HO-1 ELISA; Enzo Life Sciences, Farmington, NY; ADI960071).

### Calculations and Statistics

All values are presented as means ±1 SEM. Statistical comparisons between two groups were made by unpaired Student’s t test. Comparisons between left and right kidneys from a given group of mice were performed by paired Student’s t test. Statistical significance was judged by a p value of <0.05.

## Results

### Variable Unilateral Renal Ischemia Times/18 hr Reflow

**Figure 1:**
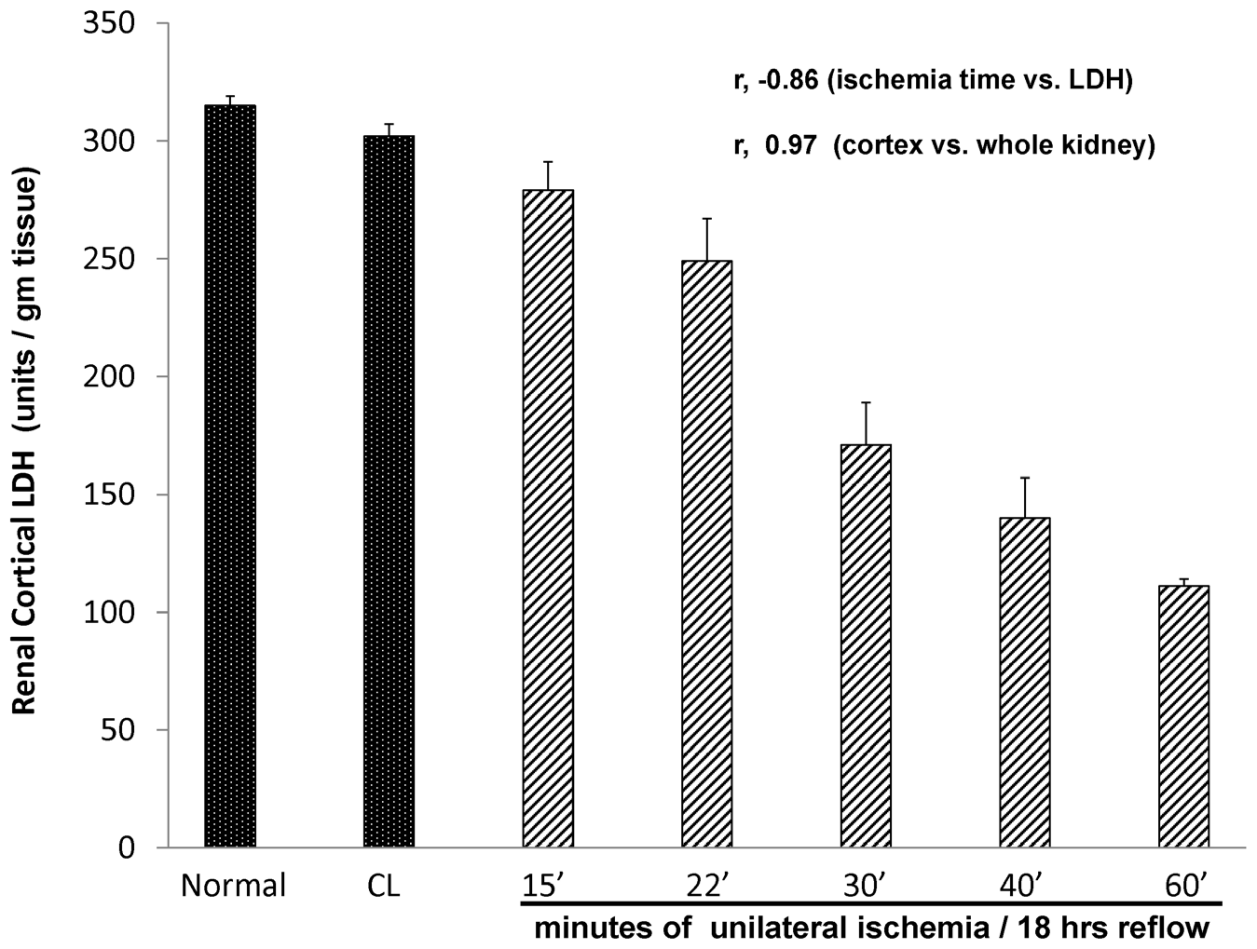
Renal cortical LDH concentrations in normal mice, and in mice 18 hrs following 15, 22, 30, 40, or 60 min of left renal ischemia. CL = contralateral (right uninjured kidneys). A steep stepwise reduction in renal cortical LDH concentrations was observed with increasing ischemia times (r, −0.86). Whole kidney LDH concentrations were also measured and an essentially perfect correlation with renal cortical LDH concentrations was observed (r, −0.97).

As shown in Fig. 1, stepwise increases in left unilateral ischemia times caused dramatic reciprocal reductions in renal cortical LDH content (ischemia times vs. LDH content, r, −0.86, p<0.001). With the longest ischemia time tested (60 min), LDH fell to just 33% of control values. Of note, no decrease in LDH content was observed in the contralateral (CL) kidneys obtained from these unilateral ischemia experiments (NS vs. values seen in normal kidneys). This indicates that the LDH reductions in the post-ischemic kidneys were a direct result of ischemic damage, rather than arising from surgical stress imposed by the ischemia protocols. An extremely tight correlation was observed between renal cortical vs. whole kidney LDH content (r, 0.97).

**Figure 2:**
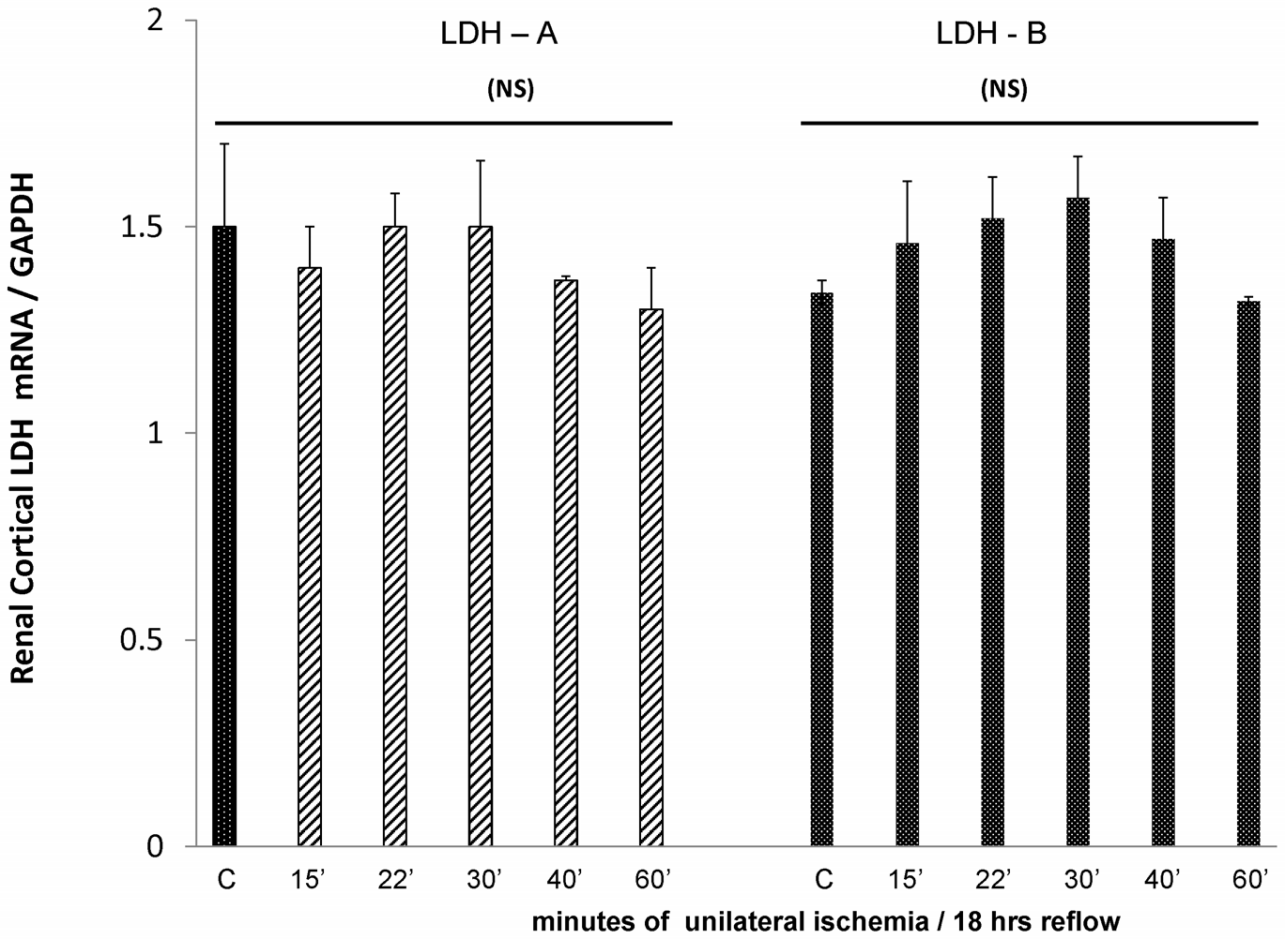
Renal cortical concentrations of LDH-A and LDH-B mRNAs in kidneys subjected to graded renal ischemia/18 hrs reperfusion. No significant changes in either mRNA were observed, implying that the falling renal cortical LDH levels were not a reflection of decreased LDH gene transcription/protein synthesis. Values are expressed as ratios to simultaneously obtained GAPDH product.

As shown in Fig. 2, none of the ischemia protocols induced a significant decrease in the levels of either LDH-A or LDH-B mRNAs. This implies that the stepwise LDH protein reductions depicted in Fig. 1 were due to LDH protein loss, rather than a suppression of LDH synthesis. As expected, due to the presence of one normal kidney, none of the unilateral ischemia protocols produced BUN elevations at 18 hrs (controls 25±4; 28±5, 23±2, 22±1; 24±2; 27±3 mg/dL for 15, 22, 30, 40, and 60 min ischemia, respectively).

### Variable Bilateral Ischemia Times/18 hr Reflow: Kidney, Urine and Plasma Assessments

**Figure 3:**
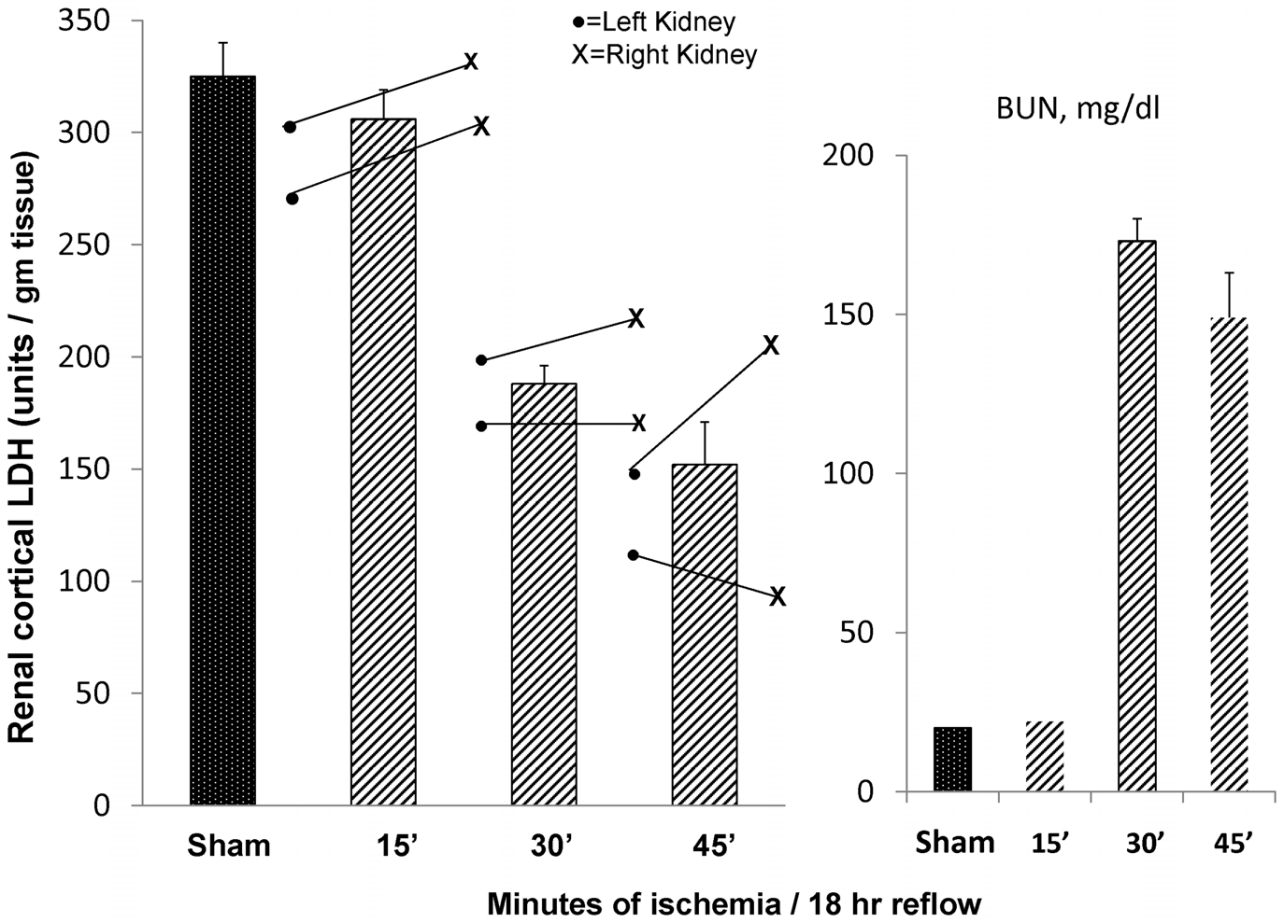
Renal cortical LDH concentrations in sham operated mice and in mice subjected to 15, 30, or 45 min of bilateral ischemic injury. The vertical bars reflect the mean ±1 SEM for the left and right kidneys at any given ischemic time. The dots and Xs reflect individual left vs. right kidney values. Four depicted results are noteworthy: 1) as with unilateral ischemia, a steep inverse dose-response relationship between renal cortical LDH and ischemia times was observed; 2) despite the equal amount of left vs. right ischemia in any of the three groups, different degrees of LDH reduction in left vs. right kidneys were apparent. These are differences in injury severity that would be missed by measuring BUN or creatinine concentrations; 3) 15 min of ischemia did not raise BUN levels, but a small and significant reduction in LDH content was observed (p<0.015). This implies greater sensitivity of LDH vs. BUN assay; and 4) 45 min of ischemia evoked slightly less, rather than greater, BUN elevations than did 30 min of ischemia, indicating the lack of sensitivity of BUN as a marker of severe tubular injury (where maximal rates of urea synthesis can limit BUN increases). In contrast, LDH assay provided a discrimination in injury severity (p<0.015 for 30 min vs. 45 min LDH levels).

Comparable to unilateral ischemia, increasing lengths of bilateral ischemia led to stepwise decreases in renal cortical LDH concentrations (ischemia times vs. tissue LDH; r, −0.90; see Fig. 3, left). Despite the fact that, in any given mouse, an identical length of ischemia was imposed on the left vs. the right kidney, the degrees of LDH reductions were not identical, implying that slightly different levels of ischemic damage existed (which would certainly be missed if relying on BUN or plasma creatinine assessments). Of further interest, 30 min and 45 min of ischemia induced the same approximate BUN increases (Fig. 3, right), despite the difference in underlying injury induced by different ischemia times. This undoubtedly reflects the fact that the height of BUN elevations at 18 hrs post ischemia was limited by the amount of urea that could be generated in a fixed amount of time. In contrast, tissue LDH assay detected significantly lower levels in the 45 min vs. the 30 min ischemia groups (p<0.05). This underscores greater LDH vs. BUN sensitivity for detecting the extent of severe renal damage.

**Figure 4:**
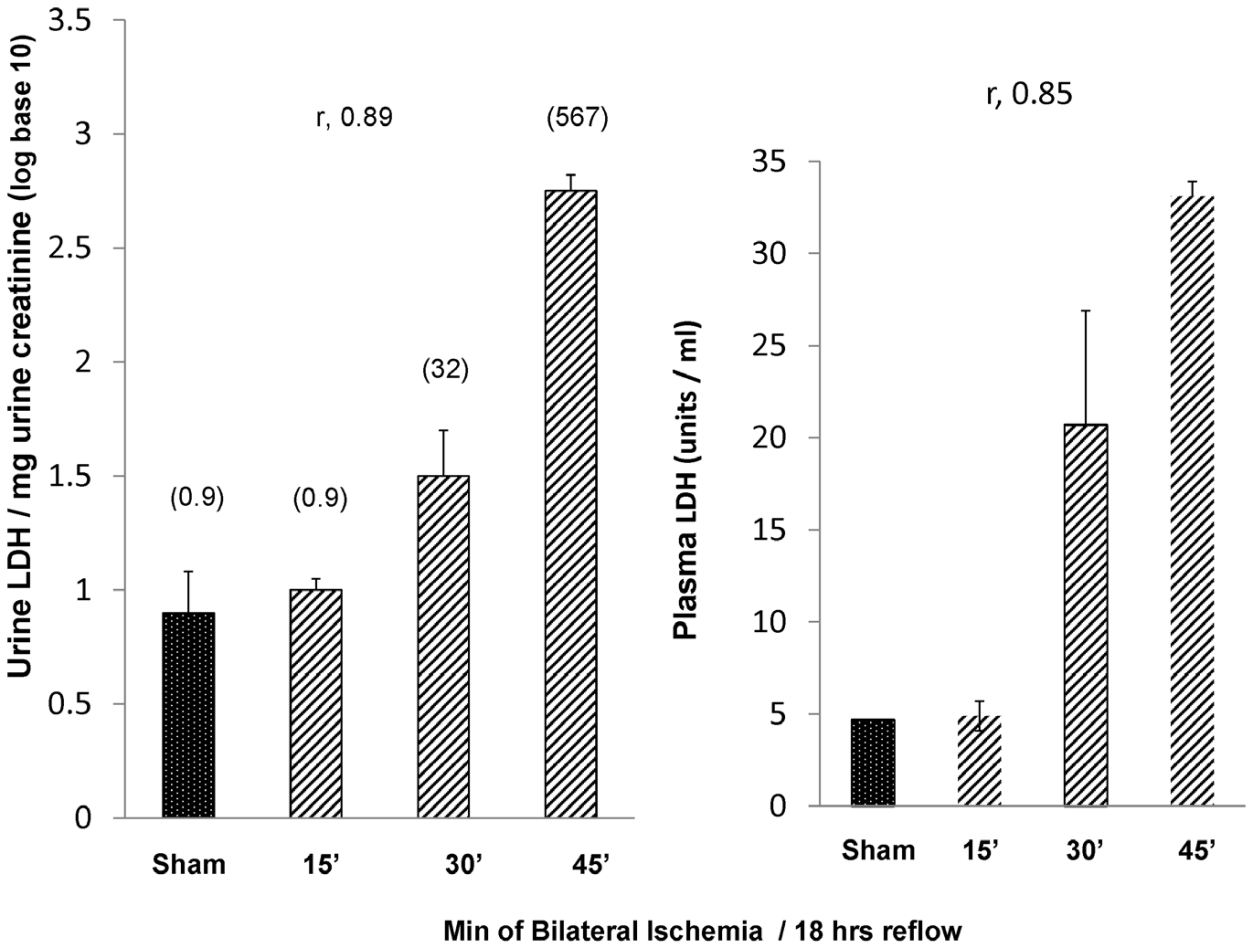
Urine and plasma LDH values following 15, 30, and 45 min of ischemia (data obtained from the mice depicted in figure 3). Corresponding with the declining renal cortical LDH values with increasing ischemia times shown in figure 3 were stepwise, massive increases in urinary and plasma LDH values. The urine values are presented as log base 10 for ease of graphing. The numbers above the bars represent absolute mean values.

The stepwise increases in bilateral ischemia times depicted in Fig. 3 led to marked stepwise increases in urinary LDH/creatinine ratios (Fig. 4, left panel; presented as log base 10 values for ease of graphing). The absolute mean LDH/creatinine ratio values are given within the parentheses at the top of each bar. With the 45 min ischemic challenge, the absolute LDH/creatinine ratio rose from a baseline value of 0.9 to 567 units LDH/mg urine creatinine. Similarly, stepwise increases in plasma LDH values were observed with increasing ischemia times (Fig. 4, right panel). These results imply that increased urinary LDH excretion and LDH entry into the systemic circulation were the likely causes of the falling renal LDH content.

### Variable Unilateral Ischemia/2 hrs Reflow

**Figure 5:**
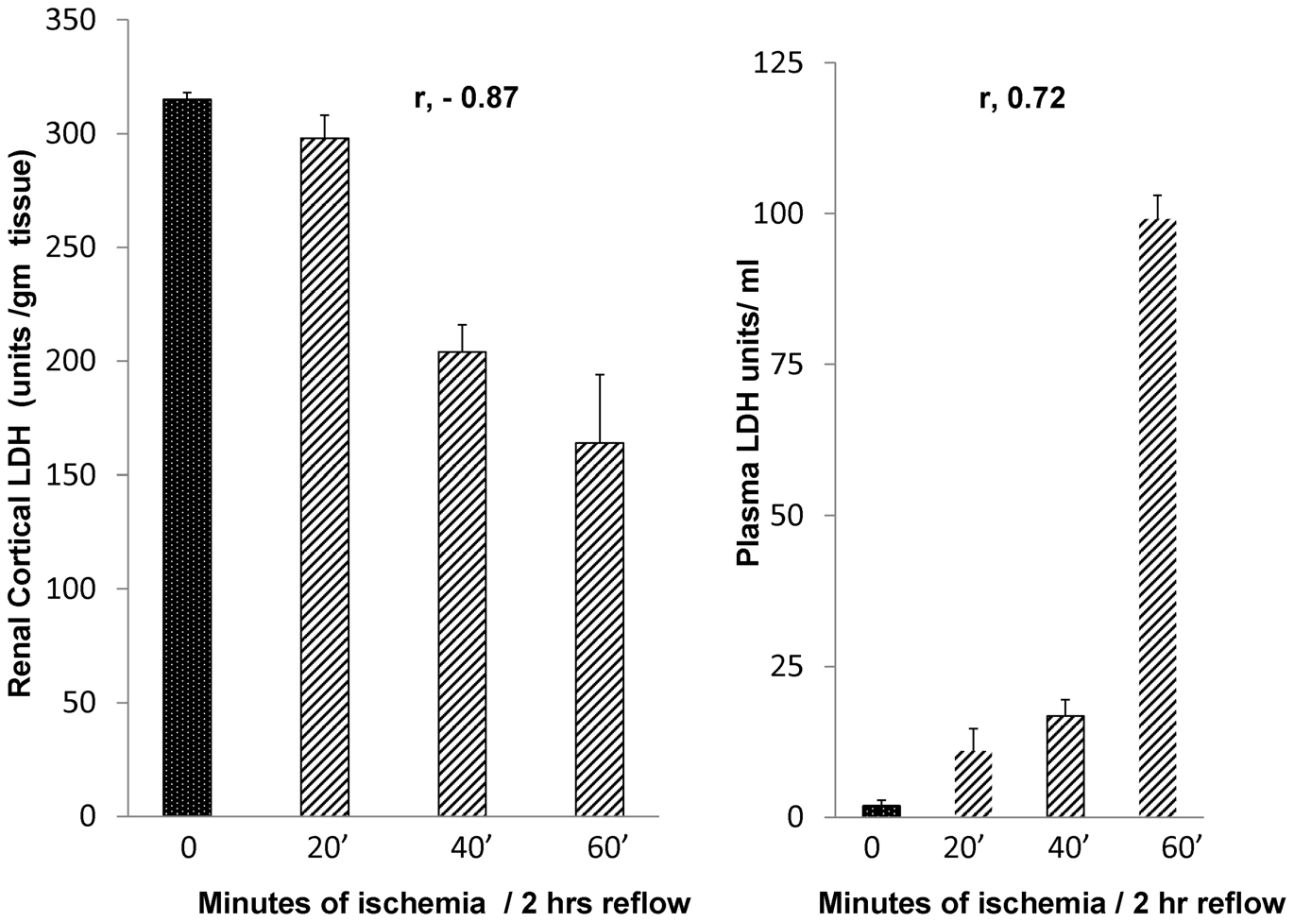
Loss of renal cortical LDH with increasing ischemia times, as documented within just two hours of the induction of ischemic/reperfusion injury. By just 2 hrs post ischemia, marked and stepwise renal cortical LDH reductions were apparent. These losses were associated with massive plasma LDH elevations. Thus, these data indicate that renal cortical LDH content can detect early ischemic injury, and that at least some of the LDH reductions are a result of efflux into the systemic circulation.

Within just 2 hrs of vascular reflow, steep cortical LDH reductions were apparent (Fig. 5, left) which corresponded with the length of the initial ischemic episode (r, −0.87). Furthermore, stepwise increases in plasma LDH concentrations were observed, consistent with early renal venous LDH efflux (Fig. 5, right).

### Assessment of whether Non Ischemic Forms of AKI can be Detected by Decreases in Renal Cortical LDH Concentrations

**Figure 6:**
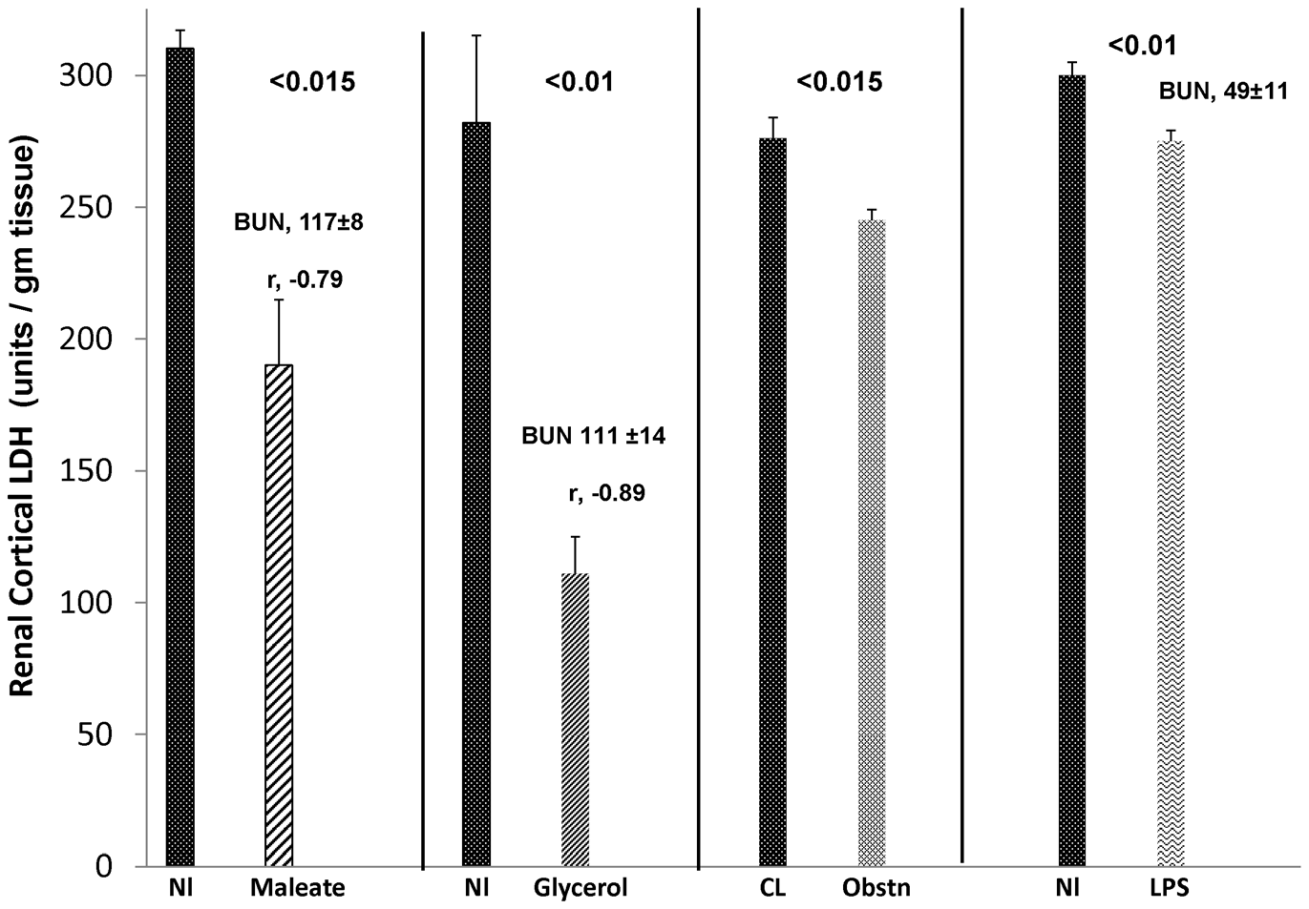
Renal cortical LDH reductions following maleate and glycerol induced AKI. Both AKI models evoked marked renal cortical LDH decrements. The degree of LDH reductions strongly and inversely correlated (r values above bars) with the extent of renal injury, as denoted by the concomitant 18 hr BUN elevations. In contrast, unilateral ureteral obstruction (obstn; CL = contralateral kidney) and endotoximia (LPS), neither of which cause overt tubule necrosis, caused relatively trivial LDH reductions. Nl = values in normal kidneys.

As shown in Fig. 6, both maleate and glycerol AKI models induced severe renal injury, as denoted by marked increases in BUN concentrations by 18 hrs post injections (values given above the bars). Corresponding with this injury were approximate 50% reductions in renal cortical LDH content. Notably, strong inverse correlations between degrees of BUN elevations vs. residual renal cortical LDH levels were observed (r, −0.89 and r, −0.79 for glycerol and maleate models, respectively).

The glycerol AKI model produced a dramatic increase in plasma LDH concentrations (58±4 units/ml vs. 2±1 for controls), undoubtedly due to the fact that glycerol induces both severe muscle injury and intravascular hemolysis (each of which release LDH). However, despite this increase in circulating LDH, glycerol still evoked dramatic renal cortical LDH reductions. This is consistent with the concept that LDH, with a molecular mass of ∼150 kDa, coupled with AKI induced GFR reductions, would not be expected to undergo significant glomerular filtration. Thus, it would not be expected to significantly impact renal cortical LDH determinations.

In contrast to glycerol and maleate, unilateral ureteral obstruction (obstn) induced only a trivial reduction in renal LDH content (Fig. 6). This is consistent with the fact that at 18 hrs post induction, ureteral obstruction does not induce overt tubular necrosis. Furthermore, although endotoxemia induced modest azotemia (61±8 mg/dl; vs 22±1 mg/dl for control mice), it had only a minimal effect on renal cortical LDH levels (again consistent with the fact that this is a hemodynamic form of AKI with minimal tubule necrosis). Unilateral ureteral obstruction did not significantly increase BUN concentrations (24±2 mg/dl).

### Renal Cortical NGAL Assessments; Comparison to Renal Cortical LDH Results

**Figure 7:**
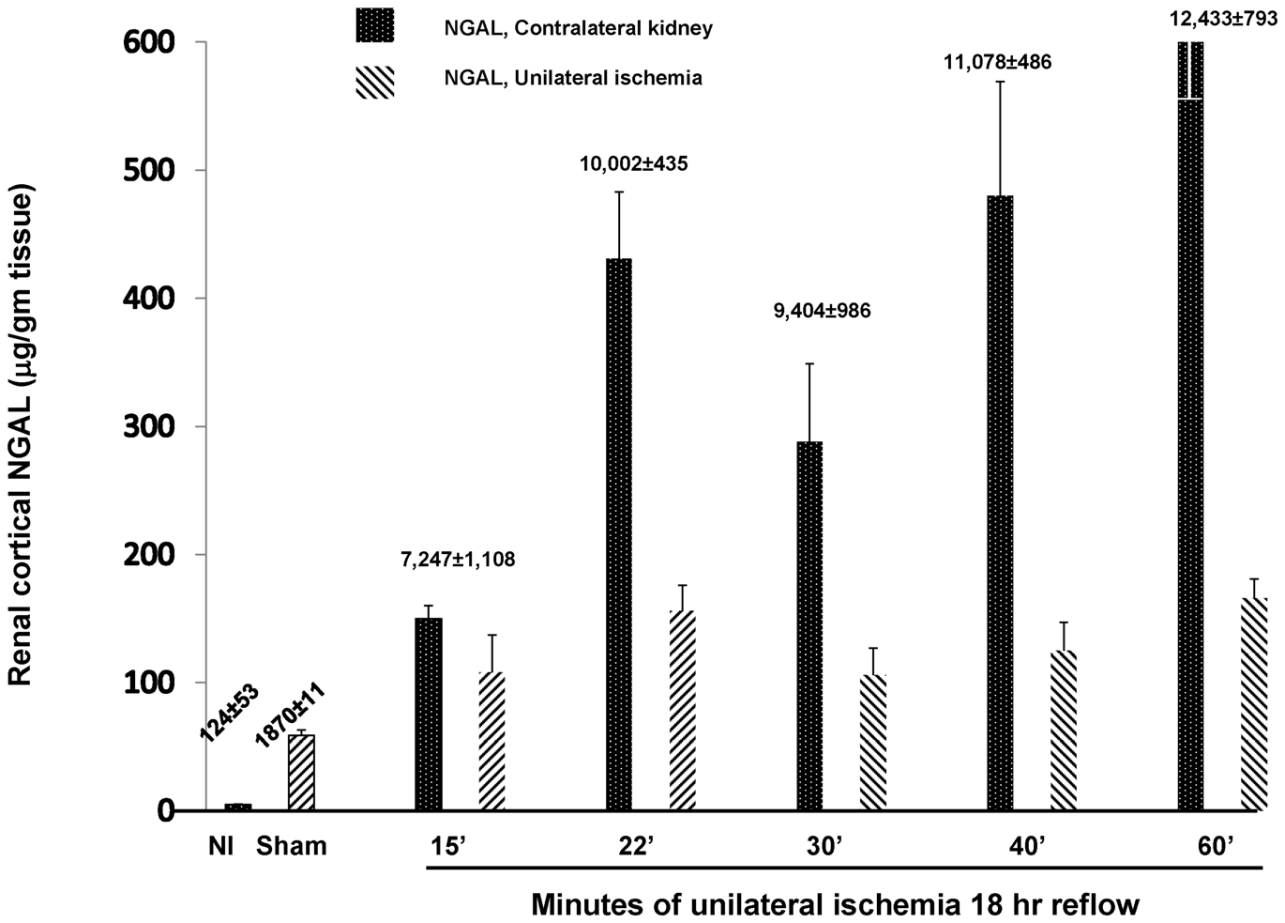
Renal cortical NGAL protein levels following graded left renal ischemia/18 hrs reflow in both the post ischemic and contralateral kidneys. The corresponding plasma NGAL concentrations (ng/ml; mean ±1 SEM) are presented above the bars. As shown at the far left, sham surgery caused a marked increase in renal cortical NGAL concentrations, despite the lack of direct renal injury. In the post ischemic kidneys only small additional NGAL increases were observed. The post ischemic increases were comparatively trivial to those in the contralateral uninjured kidneys (see text).

As shown in Fig. 7, NGAL concentrations were markedly elevated in renal cortical extracts obtained from sham operated mice, compared to values seen in kidneys from normal (Nl) mice (42±1 vs. 1.3±0.2 µg/gm tissue, respectively; p<0.001). Renal ischemia induced only modest further cortical elevations, and the post ischemic values correlated poorly (r, −0.21) with the length of renal ischemia that had been imposed. In sharp contrast, massive NGAL increases were observed in contralateral (uninjured) kidneys (∼2–4 times higher than values seen in their ischemic counterparts). Marked plasma NGAL levels were also observed (ng/ml; see numbers above the bars in Fig. 7). Given NGAL’s low molecular weight (∼25kDa), these high circulating NGAL levels presumably underwent glomerular filtration, and thus, caused the marked increases in contralateral kidney NGAL levels.

### Testing the Potential Research Utility of Renal Cortical LDH Content

#### Renal ischemia reperfusion

**Figure 8:**
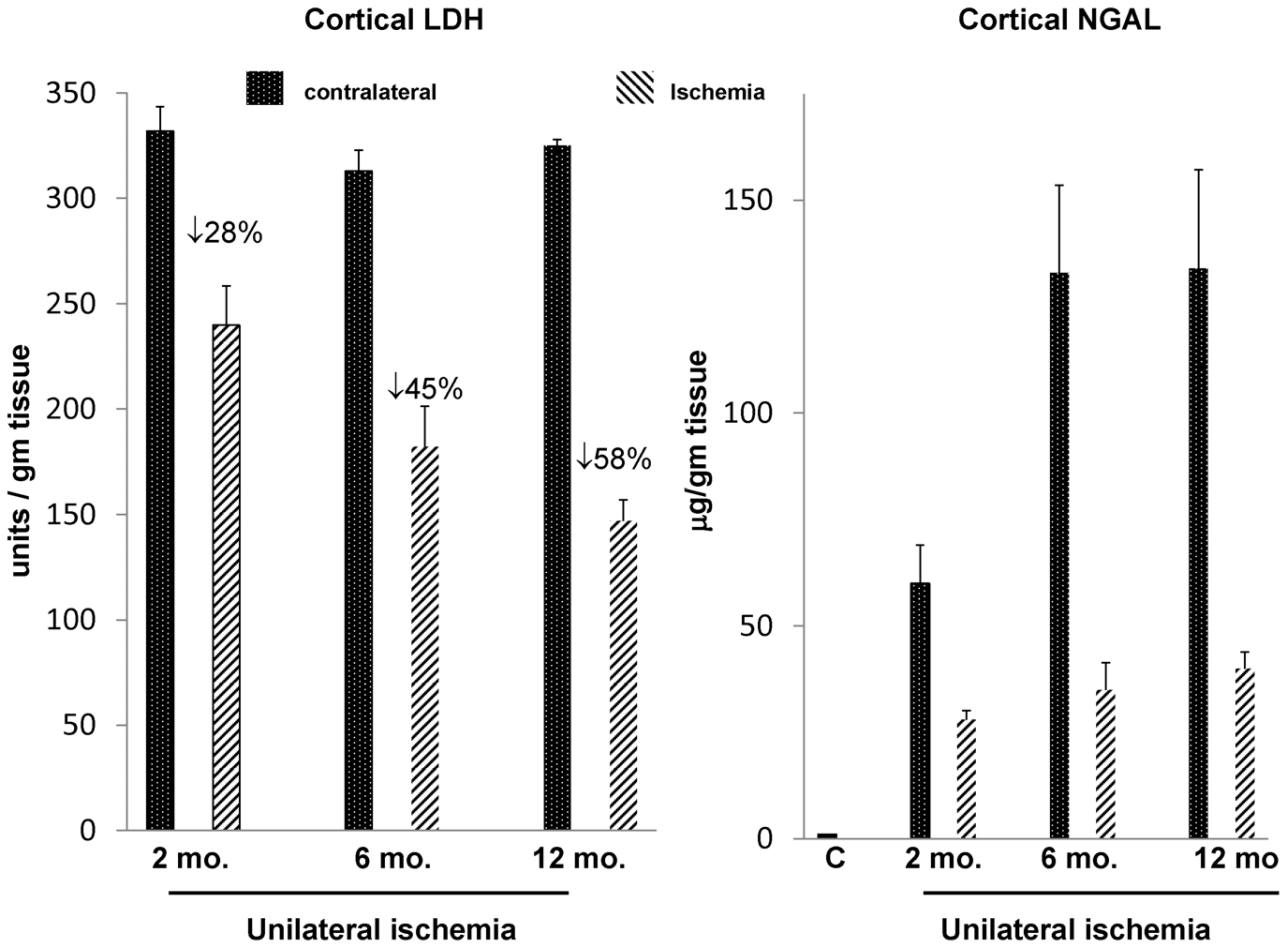
Renal cortical LDH and NGAL levels following 30 min of unilateral ischemia in 2 month, 6 month, and 12 month old mice. With increasing age, increasing renal tubular damage was apparent by stepwise decreases in renal cortical LDH levels. The % reduction in LDH at each age was determined by comparing the LDH values in the contralateral (uninjured) kidney vs. the 30 min post ischemic kidney. Of note, no differences in contralateral LDH values were observed with increasing age. In contrast to LDH, post ischemic NGAL levels correlated poorly with ischemia times (r, −0.21), and they were relatively “dwarfed” by the increases seen in the contralateral kidneys.

Mouse age (2, 6, 12 months) did not affect the amount of LDH in non-ischemic (contralateral) kidneys. However, with increasing age, kidneys subjected to ischemic- reperfusion injury manifested stepwise LDH reductions (Fig. 8, left). Thus, by comparing the contralateral (right kidney) vs. the post ischemic (left) kidney LDH values, the % LDH reductions following ischemia-reperfusion injury were 28%, 45%, and 58% in the 2 month, 6 month, and 12 month old mice, respectively (indicating increasing tubule damage with increasing age).

In contrast, NGAL concentrations in post-ischemic kidneys revealed only a slight and non-significant, trend towards progressively higher values with advancing age (Fig. 8, right). However, in every case, the NGAL values were *dramatically lower, not higher*, in the post- ischemic kidneys vs. their contralateral (uninjured) counterparts. Dramatic plasma NGAL elevations were also observed (controls, 125±53; 6661±711; 9277±1018, and 9804±1044 ng/ml for 2 month, 6 month and 12 month mice). With higher GFRs in contralateral vs. post ischemic kidneys, greater NGAL filtration/renal uptake likely accounted for the higher renal NGAL levels in the uninjured vs. injured kidneys.

**Figure 9:**
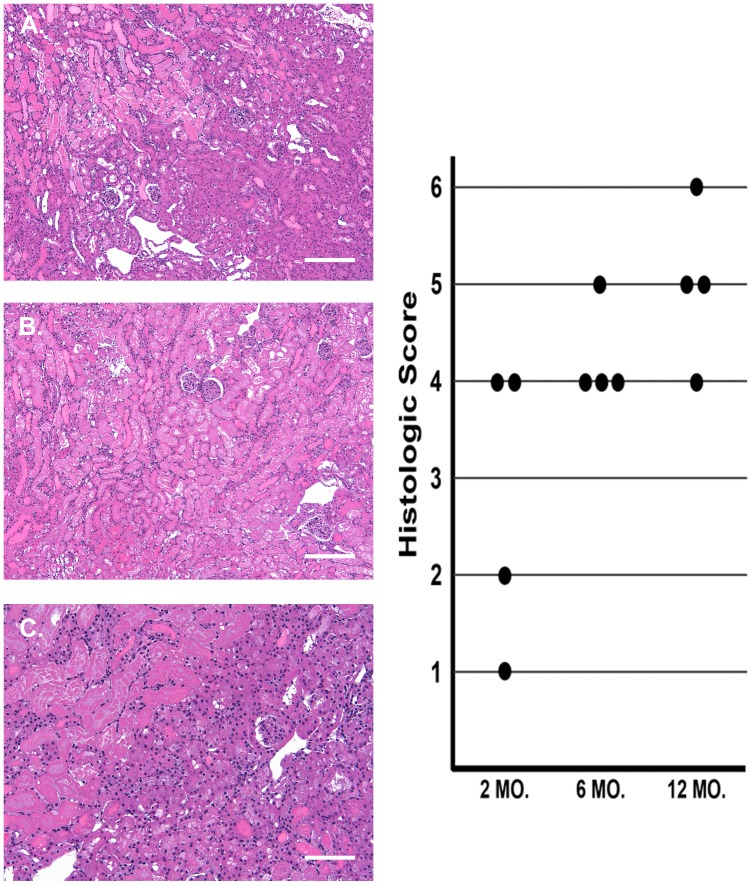
Renal photomicrographs of post ischemic injury in kidneys from 2 month vs. 12 month old mice, and corresponding histopathologic scores. Panel A depicts a 2 month old post ischemic kidney, showing extensive necrosis in the outer medullary stripe, but with relative cortical sparing (scale bar, 200 microns). Panel B depicts a 12 month post ischemic kidney which demonstrates widespread outer medullary/cortical necrosis (bar, 200 microns). Panel C is also from a 12 month old mouse which demonstrates the patchy nature of ischemic tubular necrosis (severe in upper left; essential normal preservation of morphology in lower right; bar, 100 microns). Thus, the point to be illustrated is that in any given kidney, severe vs. minimal histologic injury can be seen. Semi-quantitative assessments of injury reflected a clear trend to higher injury scores with increasing age, but that significant overlap exists. Thus, LDH assessments appear to provide a more quantitative/less subjective assessment of renal damage.

Figure 9, right, depicts the semi-quantitative histologic injury scores obtained from blinded grading of the severity of histologic injury. There was a clear trend towards higher injury scores with increasing mouse age. However, there was considerable variation in the scores, particularly in the 2 month old mice. Furthermore, each of the three groups showed at least some 4+ scores, and there was almost total overlap of the scores between the 6 month and 12 month mice. Thus, much clearer definition of differences in injury severity was achieved with tissue LDH vs. histologic or NGAL assessments. Examples of the histologic injury are presented. The two month mice showed prominent necrosis, mostly in the outer medullary stripe (OMS; Fig. 9, Panel A), whereas the 6 month and 12 month (Panel B) mice showed widespread but patchy necrosis in both the OMS and renal cortex. The patchy nature of the necrosis, even in the 12 month mice, is depicted in Panel C, which shows extensive necrosis (upper left) abutting an area of near normal histology (lower right).

#### Glycerol model of AKI

As presented in Table 2, 2 month and 12 month old mice showed the same severity of glycerol- induced AKI, as assessed by BUN and plasma creatinine elevations over baseline. In agreement with these results, virtually identical renal cortical LDH reductions were observed in the two groups. Because of previous overwhelming evidence that heme oxygenase 1 (HO-1) is a critical cytoprotectant against heme protein- induced AKI [19]–[21], HO-1 protein levels were measured in additional 2 month and 12 month old mice that had not been subjected to glycerol injection (n, 3 each). Under basal conditions, the HO-1 protein values were twice as high in the 12 month vs. 2 month old mice (Table 2). Similarly, the HO-1 mRNA at baseline tended to be higher, albeit not statistically so, in the older vs. the younger group (consistent with the higher HO-1 protein levels). Thus, the higher baseline HO-1 levels may explain for why no greater severity of glycerol- induced AKI was apparent in the older mice. Both the young and old mice mounted marked and approximately equal HO-1 protein and mRNA increases in response to glycerol. Recently, it has been suggested that differences in HO-2 mRNA induction may impact heme protein nephrotoxicity [16]. However, HO-2 mRNA levels did not differ at baseline, and no statistically significant HO-2 increases were observed post glycerol injection (consistent with the fact that HO-2 is largely a constitutively expressed, rather than an inducible enzyme [23], [24]. Plasma HO-1 levels markedly, and equally, increased in both 2 and 12 month mice following glycerol injection. Given that plasma HO-1 elevations can serve as a biomarker of AKI severity [24], the identical plasma HO-1 elevations in the 2 month vs. the 12 month mice are in agreement with the equal BUN and plasma creatinine increases, and equal cortical LDH reductions. Thus, all of the available data indicated comparable injury severity. This provides further evidence for the validity of renal cortical LDH assessments.

**Table 2:** Renal injury parameters 18 hrs following glycerol injection into 2 vs. 12 month old mice.

	2 months: baseline	2 months: post glycerol	12 months: baseline	12 months: post glycerol
**BUN**	20±3	135±7	18±3	103±17
**Creatinine**	0.4±0.1	0.8±0.2	0.4±0.2	1.0±0.2
**Tissue LDH**	325±10	214±8	310±15	219±11
**HO-1 (protein)**	1.6±0.4	7.9±0.8	3.3±0.3**	8.8±0.7
**HO-1 mRNA**	2.1±0.1	20.2±3.6	3.2±0.3	27.2±4.6
**HO-1 plasma**	0.0±0	24.9±3.0	0.0	23.8±7.8
**HO-2 mRNA**	6.4±0.3	8.4±0.8	6.3±0.3	10.4±1.0

Both groups of mice mounted marked and approximately equal BUN (mg/dl) and creatinine (mg/dl) increases in response to glycerol injection. Renal tissue LDH levels significantly fell (<0.01) in both groups of mice, and to almost identical degrees. HO-1 protein levels were twice as high at baseline in the 12 month vs. the 2 month mice (**p<0.01). Although baseline HO-1 mRNA values also appeared to be higher in the 12 month vs. 2 month group, these results were not statistically different (p, 0.1). Both groups manifested marked and approximately equal renal HO-1 mRNA and protein increases in response to glycerol. Marked and equal post glycerol increases in plasma HO-1 levels were also observed. Finally, HO-2 mRNA levels did not significantly differ between the two groups either at baseline or at 18 hr post glycerol injection.

## Discussion

It is widely recognized that experimental studies of AKI are highly dependent on the availability of precise quantitative markers of proximal tubule injury. However, as reviewed in the Introduction of this article, currently utilized markers of tubular injury, including indirect assessments of GFR (i.e., BUN, creatinine), levels of stress protein induction (e.g., NGAL, HO-1), and renal histologic assessments, each have major limitations. In contrast, in cell culture experiments, precise injury markers are available, due to direct access to, and the ability to perform direct assessments on, targeted cells. A classic example is calculating LDH release from the cytosol into cell culture medium. However, to the best of our knowledge, no studies have critically evaluated the potential utility of LDH release as a quantitative marker of in vivo ischemic or toxic renal injury.

Upon first consideration, one might readily dismiss the possibility that renal cortical LDH assessments might have utility in this regard. For example, LDH is released from multiple extrarenal organs in response to diverse forms of injury, and resulting increases in circulating LDH levels could potentially obfuscate interpretation of kidney LDH levels. Alternatively, although renal infarction is known to raise plasma LDH levels [25], it is unclear whether less catastrophic forms of AKI permit LDH release from cells, with subsequent prompt and quantitative renal exodus. For example, LDH released into the renal interstitium or into tubular luminal casts could potentially be trapped within these compartments, thereby preventing acute declines in renal cortical or whole kidney LDH content. Given these considerations, this study was performed to ascertain whether renal LDH declines occur with AKI, and if so, would they have utility as a marker of renal injury severity.

To first address this question, graded unilateral ischemia was imposed on renal tissues and by 18 hrs later, a surprisingly steep inverse correlation between ischemia times and renal cortical LDH levels were observed (r, −0.86). Of note is that the contralateral kidneys from these experiments manifested no LDH reductions (compared to normal kidney values), thereby indicating that the post- ischemic LDH declines were, indeed, due to ischemic cell injury, rather than a general response to surgical stress. To expand on these initial observations, graded degrees of bilateral ischemic injury were imposed and again a near perfect (r, −0.90) correlation between ischemia times and renal cortical LDH declines was observed. Finally, to ascertain whether tissue LDH reductions in response to ischemia could be an early marker of tubule injury severity, renal cortical LDH reductions were assessed at two hrs post ischemia, and even at this early time point, a striking inverse correlation between renal cortical LDH content and renal ischemia times was observed. Of interest was that 45 min of bilateral ischemia did not induce greater BUN elevations than did the 30 bilateral ischemic challenge. This undoubtedly reflects the fact that with severe AKI, the degree of BUN elevations in limited by urea generation rates. In contrast to BUN, the % of renal cortical LDH reductions with 30 vs. 45 min of ischemia were significantly different (53% vs. 68% declines respectively). This clearly indicates that in the presence of severe AKI, tissue LDH assessments are superior to BUN determinations for gauging renal injury severity. The bilateral ischemia experiments also make the point that despite the fact that the same ischemia times were applied to both left and right kidneys (either 15, 30, or 45 min), modest differences in the extent of left vs. right kidney LDH reductions were observed. This was not unexpected, given that renal artery occlusion induces variable degrees of renal injury despite a constant clamp time. Obviously, left vs. right kidney differences in injury severity cannot be detected by BUN or creatinine assessments, given that the latter reflect total kidney function. In situations in which both kidneys are to be used for assessments of injury pathways, it is critical to know the exact amount of injury induced in each sampled kidney. Such information can be provided by LDH assessment.

A key question that arises from the above experiments is what is the mechanism for the falling renal LDH content in response to AKI? Three possibilities are as follows: first, that LDH is released from the renal cytosol into the urinary space and the interstitial compartments, from where it can be eliminated into final urine and plasma, respectively. The second possibility is that an acute suppression of LDH synthesis occurs, which either causes or contributes to tissue LDH reductions. The third potential mechanism is that LDH is rapidly degraded within the cytosol, thereby lowering intrarenal concentrations. The available evidence strongly points to the first possibility, given that marked, stepwise increases in both urinary and plasma LDH concentrations were observed following ischemic renal damage (Fig. 4). Indeed, that rapid LDH entry into plasma can occur is indicated by the observation that increased plasma LDH levels were observed within just two hrs following ischemic renal damage (Fig. 5). To evaluate the second possibility, i.e., that decreased LDH synthesis might contribute to falling renal LDH content, we measured LDH-A and LDH-B mRNAs as surrogate markers of LDH synthesis, and no decrements were observed. Hence, decreased LDH production seems unlikely. We cannot exclude the third possibility: that increased LDH proteolysis occurs within the kidney which then contributes to depressed LDH levels. However, arguing against this possibility is that in a variety of ATP depletion injury and nephrotoxic cell culture experiments, LDH is lost from the cytosol into cell culture media, but the total LDH content (cell pellets+cell culture media) is preserved (11, 12). This implies that AKI does not induce marked LDH proteolysis.

To assess whether renal cortical LDH assessments might have utility in non ischemic forms of renal injury, we studied the glycerol, maleate, unilateral ureteral obstruction, and endotoxemic models of AKI. With both glycerol and maleate, ∼50% renal cortical LDH reductions were observed, and in both cases, impressive inverse correlations between renal cortical LDH content and BUN concentrations were observed (e.g., r, −0.89 between post glycerol BUN and tissue LDH levels). Conversely, in the case of unilateral ureteral obstruction or endotoxemia, only trivial LDH reductions occurred. This was as expected, given that neither of these 18 hr AKI models induces substantial proximal tubule necrosis. Hence, LDH reductions would not be expected to occur. Finally, a critical point emerged from the studies of glycerol– induced AKI. This model induces marked muscle injury and intravascular hemolysis, thereby markedly increasing plasma LDH concentrations. However, despite these marked increases, renal cortical LDH content still fell by ∼50%. This indicates that high circulating LDH levels do not negate the utility of renal LDH assessments. This undoubtedly reflects the fact that LDH, with a molecular weight of ∼150,000, would not be expected undergo glomerular filtration and hence, renal uptake. This is a critical point because many forms of experimental ARF are associated with extra-renal injury (e.g., rhabdomyolysis, cecal ligation and puncture), potentially resulting in plasma LDH increases that might otherwise compromise renal cortical LDH assessments.

The limitations of BUN, creatinine, and renal histology as precise indices of tubular injury have led to investigations as to whether levels of “stress protein” induction might serve as alternative quantitative injury markers. However, as previously alluded to, stress protein levels reflect production by surviving, not necrotic/apoptotic, renal tubular cells. Furthermore, stress proteins can be generated at extra-renal sites, and due to their low molecular weights, they gain ready renal access. Our assessments of renal cortical NGAL in the present study underscore these limitations. First, the employed ischemia-reperfusion surgical protocol, as well as sham surgery, induced massive plasma NGAL increases. With presumed glomerular filtration, this resulted in the paradox of much higher NGAL levels in contralateral (non ischemic) kidneys vs. their ischemia– damaged counterparts. Second, even when restricting NGAL evaluation to the post-ischemic kidneys, an almost flat dose – response curve between ischemia times vs. post ischemic NGAL concentrations was observed. Thus, at least in the case of NGAL, a variety of factors, in addition to injury severity, must impact its expression. In contrast, renal LDH loss does not appear to suffer from these limitations, given that its high molecular weight retards its filtration, and its rapid egress from the kidney presumably reflects a far less complex biologic process than stress protein induction.

Given the apparent utility of renal cortical LDH levels as an accurate marker of renal tubular injury, we decided to use this technique to address a previously unanswered question that has persisted in the literature. We, as well as others, have noted that increasing age imparts increasing renal susceptibility to ischemic and toxin mediated AKI [16]–[19]. However, it has remained unclear as to whether the greater GFR reductions in aged vs. young rodents reflect differences in glomerular hemodynamics vs. differences in tubule susceptibility to injury. Indeed, in our initial report of this phenomenon, we observed significantly greater post- ischemic GFR reductions in old vs. young rats despite comparable morphologic damage [18]. Therefore, to gain new insights into this issue, in the present study, 2 month, 6 month, and 12 month old mice were subjected to ischemic AKI and then renal cortical LDH assessments and renal histologic analyses were performed. Increasing age led to striking stepwise reductions in renal cortical LDH content, consistent with increasing renal tubular damage. In contrast, although renal histology confirmed increasing injury with advancing age, substantial overlap in individual histologic scores was observed, particularly in the 6 vs. 12 month groups. Thus, the clear separation of injury severity by LDH assessments, but not by renal histology, serves to underscore the sensitivity and utility of renal cortical LDH assessments as a direct gauge of tubular damage.

In contrast to ischemic AKI, no difference in the severity of glycerol- induced AKI was noted with advancing age, as indicated by essentially identical degrees of renal cortical LDH loss from 2 month and 12 month old mice. That BUN and creatinine levels were also highly comparable in the 2 month vs. 12 month mice supports the validity of the LDH assessments. Of note, these findings appear to contradict those in a recent publication by Nath et al who reported that increasing age confers increasing renal susceptibility to heme protein toxicity [16]. In that study, it was concluded that this difference was due to blunted HO-2 gene induction in old vs. young mice. However, in our study, HO-2 mRNA was not significantly induced by glycerol in either group (consistent with the traditional view that HO-2, unlike HO-1, is a constitutively expressed, vs. an inducible, enzyme) [23], [24]. Furthermore, in our study, baseline HO-1 protein levels were twice as high in old vs. young mice, which may well explain why we found that old and young mice had comparable susceptibility to the glycerol induced AKI. Conversely, in Nath’s study, HO-1/HO-2 protein levels were not assessed. But whatever the reason for our discrepant results with those recently reported [16], the key point in our experiment is that the extent of post glycerol renal cortical LDH loss was congruent with the BUN and plasma creatinine concentrations, further validating the utility of LDH assessments.

An intriguing question is whether the present findings of marked renal cortical LDH depletion post AKI might impact subsequent energy generation (e.g., during a post- AKI recovery period, or with possible superimposed renal insults). For example, it seems possible that with a marked decrease in tissue LDH, the bidirectional flow of pyruvate - lactate could be compromised. Given that pyruvate → lactate conversion is the dominant source of anaerobic ATP production, and that lactate→ pyruvate conversion eventually allows pyruvate entry (via acetyl CoA formation) into the Kreb’s cycle, tissue LDH depletion could have potential effects on both anaerobic and aerobic energy metabolism. Thus, while the present study has focused on the potential value of renal cortical LDH measurement as a marker of AKI severity, the findings also may have substantial pathophysiologic relevance that will require further investigation.

Finally, it should be stressed that the major limitation of cortical LDH assay as a gauge of AKI severity is that it can only be used to analyze terminal kidney samples. Although LDH is released from the kidney into the systemic circulation, it is clear that plasma LDH cannot be used as a surrogate for renal cortical LDH release due to a lack of renal specificity, as discussed above. A more promising experimental (and potentially clinical) approach might be urinary LDH analysis, given that urinary excretion appears to be a major route for renal cortical LDH elimination. However, multiple factors can impact urinary protein excretion as well as urinary enzymatic analyses. Thus, at present, ready surrogate markers of renal cortical LDH levels are not at hand. However, this shortcoming notwithstanding, the present results clearly indicate that cortical LDH analysis provides a highly quantitative, simple, inexpensive, and accurate assessment of experimental ischemic and toxic kidney injury. Given that it avoids the limitations inherent to more traditional markers of AKI severity, it could have considerable utility in future experimental AKI investigations that require precise quantitative injury assessments. The potential pathophysiologic significance of tissue LDH depletion on subsequent renal regeneration or injury responses remains a promising area future study.
